# Surgical Approach and Outcomes in Early-Stage Endometrial Cancer: A Molecularly Stratified Comparison of Open, Laparoscopic, and Robotic Surgery

**DOI:** 10.3390/medicina61122093

**Published:** 2025-11-24

**Authors:** Mohamed Abdelwanis Mohamed Abdelaziz, Siddesh Prabhulingam, Ayodele Olaleye, Ambreen Yaseen, Khaled Sabrah, Riyam Aldulaimi, Nesma Hesham, Ahmed Mohamed, Hossam Ali, Ketankumar Gajjar

**Affiliations:** 1 Department of Gynaecological Oncology, Nottingham University Hospitals NHS Trust, City Hospital, Nottingham NG5 1PB, UK; siddesh.prabhulingam@nhs.net (S.P.); ayodele.olaleye@nhs.net (A.O.); ambreen.yaseen@nhs.net (A.Y.); riyam.aldulaimi@nhs.net (R.A.); ahmed.mohamed28@nhs.net (A.M.); ketan.gajjar@nhs.net (K.G.); 2 Department of Gynaecology and Obstetrics, Queen’s Medical Centre, Nottingham University Hospitals NHS Trust, Nottingham NG7 2UH, UK; khaled.sabrah2@nhs.net (K.S.); nesma.hesham@nhs.net (N.H.); 3 Department of Gynaecology and Obstetrics, Nottingham University Hospitals NHS Trust, City Hospital, Nottingham NG5 1PB, UK; hossam.ali1@nhs.net

**Keywords:** minimally invasive surgery, endometrial cancer, surgical outcomes, molecular classification, bias control, robotic surgery, laparoscopic surgery

## Abstract

*Background and Objectives*: Previous surgical approach comparisons in endometrial cancer may be confounded by inadequate control for tumour biology—the primary driver of outcomes. This study provides the first surgical approach comparison incorporating molecular classification to control for case selection bias. *Materials and Methods*: Retrospective analysis of 512 consecutive patients with stage I–II endometrial cancer (FIGO 2009) treated with open (*n* = 83), laparoscopic (*n* = 278), or robotic (*n* = 151) surgery between 2018 and 2024. Molecular classification was available for 219 patients (42.8%) using TCGA criteria and incorporated into analyses to control for case selection bias, with molecular subtype incorporated to control for biological bias rather than as a primary endpoint. Primary outcomes included perioperative metrics and oncological safety. The primary objective was to determine whether apparent surgical outcome differences reflect genuine technique effects or case selection bias based on tumour biology. *Results*: Molecular subtype distribution varied significantly by surgical approach, with high-risk subtypes concentrated in open surgery, explaining apparent outcome differences. After controlling for molecular subtype and other confounders, minimally invasive approaches demonstrated superior perioperative outcomes: reduced blood loss (laparoscopic 129.8 mL, robotic 157.9 mL vs. open 261.4 mL, *p* < 0.001), shorter hospital stays (2.4 and 2.2 vs. 5.3 days, *p* < 0.001), and lower complications (5.7% and 6.6% vs. 21.6%, *p* < 0.001). In our cohort, recurrence-free survival showed significant differences favouring minimally invasive approaches, with 2-year RFS rates of 92.8%, 96.4%, and 100.0% (*p* = 0.008) and 3-year RFS rates of 90.4%, 95.0%, and 100.0% (*p* = 0.003) for open, laparoscopic, and robotic surgery, respectively, although robotic surgery had a shorter follow-up (median 33 vs. 42 months). Within-approach exploratory analyses revealed that p53-abnormal tumours were associated with significantly longer operative times and greater blood loss across all surgical approaches (*p* < 0.05), although complication rates did not differ significantly by molecular subtype within any approach (open *p* = 0.124, laparoscopic *p* = 0.656, robotic *p* = 0.287). Apparent surgical approach differences largely reflected appropriate case selection based on tumour biology rather than technique superiority. *Conclusions*: When controlling for tumour biology, minimally invasive approaches offer superior perioperative outcomes with equivalent oncological safety. Higher complication rates in open surgery primarily reflect the inherent morbidity of this approach and appropriate surgeon selection for high-risk cases. Within-approach analyses suggest possible molecular influences on operative parameters that warrant prospective validation. Molecular stratification is essential for fair surgical approach comparison in the contemporary era.

## Simple Summary

This study compares three surgical approaches for early-stage endometrial cancer while controlling for tumour biology—a critical factor that may bias surgical approach comparisons. By analysing 512 patients and accounting for molecular tumour characteristics that influence outcomes, we found that minimally invasive approaches (laparoscopic and robotic) offer significant advantages including less blood loss, shorter hospital stays, and fewer complications, while maintaining superior cancer control. Importantly, these advantages persisted after comprehensive bias control, while apparent differences in overall outcomes between surgical groups largely reflected appropriate case selection based on tumour biology.

## 1. Introduction

Endometrial cancer represents the most common gynaecological malignancy in developed countries, with approximately 417,000 new cases diagnosed globally in 2020 [1]. According to World Health Organization projections, endometrial cancer incidence is expected to increase by 50–60% by 2040 due to aging populations and rising obesity rates, with mortality projected to increase by approximately 45% over the same period [2]. The majority of patients (75–80%) present with early-stage disease confined to the uterus, offering excellent opportunities for surgical cures [3]. However, the optimal surgical approach—open, laparoscopic, or robotic—remains a subject of ongoing debate, with comparative studies yielding inconsistent results.

### 1.1. Evolution of Surgical Approaches

Historically, open surgery via laparotomy was the standard approach for endometrial cancer, providing excellent visualisation and access for comprehensive staging [4]. The introduction of laparoscopic techniques in the 1990s marked a paradigm shift towards minimally invasive surgery, with early studies demonstrating reduced morbidity without compromising oncological outcomes [5,6]. More recently, robotic surgery has emerged as an alternative minimally invasive approach, potentially offering advantages in complex cases through enhanced dexterity and three-dimensional visualisation [7].

Large randomised, controlled trials, including the LAP2 study and LACE trial, have established the non-inferiority of laparoscopic surgery compared to open surgery for endometrial cancer staging [8,9]. These landmark studies demonstrated reduced perioperative morbidity with laparoscopic approaches while maintaining equivalent oncological outcomes.

### 1.2. The Molecular Revolution and Its Impact on Surgical Research

The landscape of endometrial cancer research has been fundamentally transformed by the recognition of molecular heterogeneity. The landmark Cancer Genome Atlas (TCGA) study identified four molecularly distinct subtypes with markedly different prognoses: POLE-ultramutated (exceptional outcomes), mismatch repair-deficient (MMRd, intermediate), p53-abnormal (p53abn, poor), and no specific molecular profile (NSMP, intermediate) [10].

This molecular classification has proven superior to traditional histological grading for prognostic stratification and is now incorporated into international guidelines, including the 2023 FIGO staging system [11,12]. Recent international validation studies have demonstrated substantial stage shifting and prognostic divergence under molecular-integrated classification, with Matsuo et al. reporting that over 50% of stage IB tumours were upstaged to stage II under the 2023 FIGO system [13].

### 1.3. The Critical Gap in Surgical Comparison Studies

Despite this molecular revolution, no surgical approach comparison study has adequately controlled for molecular subtype distribution—a critical oversight that may confound apparent technique differences. This methodological limitation is particularly problematic because high-risk molecular subtypes (p53abn) often receive open surgery due to aggressive features, low-risk subtypes (POLE, NSMP) may preferentially receive minimally invasive approaches, and without molecular stratification, case selection bias masquerades as technique superiority.

Recent international guidelines emphasise that contemporary endometrial cancer research cannot reliably assess oncological outcomes without accounting for molecular subtypes [14,15]. However, existing surgical approach comparison studies have failed to incorporate this critical confounding factor.

While molecular classification is well-established for prognostic stratification, its application as a methodological tool for controlling bias in surgical approach comparisons remains unexplored. This study introduces a novel application of molecular subtyping—not as a prognostic endpoint, but specifically as a bias control variable to adjust for case selection in surgical comparisons. This represents a paradigm shift in surgical research methodology.

### 1.4. Study Rationale and Objectives

This study addresses the critical need for an unbiased surgical approach comparison by incorporating available molecular classification data to control for case selection bias. Our primary objectives were to determine whether apparent surgical outcome differences reflect genuine technique effects or case selection bias based on tumour biology by comparing perioperative outcomes across surgical approaches while controlling for molecular subtype bias, assessing oncological safety through molecular-stratified survival analysis, determining whether apparent surgical differences reflect technique effects or case selection, and establishing a framework for an unbiased surgical approach comparison in the molecular era. Critically, our goal was not to achieve complete molecular profiling of all patients but rather to establish proof-of-concept that molecular stratification is essential for fair surgical comparison—a goal achieved through our analysis of 219 molecularly profiled patients (42.8% of the cohort).

## 2. Materials and Methods

### 2.1. Study Design and Setting

We conducted a retrospective cohort study at Nottingham University Hospitals NHS Trust, a tertiary gynaecological oncology centre serving a population of 2.5 million in the East Midlands, United Kingdom. The study was registered with the institutional clinical audit department (Project 24-911C) and conducted in accordance with the Declaration of Helsinki and STROBE guidelines for observational studies. As a retrospective analysis of routine clinical care, formal ethical approval was not required per institutional guidelines.

### 2.2. Patient Population

**Inclusion Criteria (Patients were included if they met ALL of the following criteria):**
  - Histologically confirmed endometrial adenocarcinoma;
  - FIGO 2009 stage I–II disease (all surgeries performed before 2023 FIGO implementation);
  - Primary surgical treatment between 1 January 2018 and 31 January 2024;
  - Complete surgical and pathological data available;
  - Minimum 6 months follow-up (unless death occurred earlier).
**Exclusion Criteria (Patients were excluded if they met ANY of the following criteria):**
  - Stage III–IV disease or evidence of extrauterine spread;
  - Synchronous primary malignancies;
  - Previous pelvic radiotherapy;
  - Neoadjuvant therapy;
  - Palliative surgical intent;
  - Inadequate tissue for histological assessment.

We acknowledge that the 2023 FIGO staging system, which integrates molecular features alongside anatomical criteria, represents the current standard. Our analysis specifically addresses this limitation by incorporating comprehensive molecular profiling data where available.

### 2.3. Surgical Techniques and Approach Selection

All surgical procedures involved total hysterectomy with bilateral salpingo-oophorectomy, with lymph node assessment performed based on institutional protocols and tumour characteristics.

**Definition of Surgical Complexity:**

Surgical complexity was assessed based on multiple factors that influence operative difficulty and patient outcomes:

1. **Procedural Extent:**
  - Basic procedure: Total hysterectomy with bilateral salpingo-oophorectomy;
  - Extended staging: Addition of lymph node assessment (sentinel lymph node mapping or systematic lymphadenectomy);
  - Comprehensive staging: Addition of omentectomy and/or peritoneal biopsies for high-risk histologies.
2. **Technical Difficulty Factors:**
  - Uterine size and mobility;
  - Presence of adhesions requiring adhesiolysis;
  - Patient factors (obesity, previous abdominal surgery);
  - Intraoperative findings requiring procedure modification.
3. **Rationale for Exploring Biological Complexity:**

Beyond standard measurable factors (uterine size, adhesions, patient factors), we hypothesised that molecular subtype might independently influence surgical parameters through biological mechanisms such as:

- **Enhanced tumour vascularity in high-risk molecular subtypes potentially affecting blood loss;**
- **Tissue architectural changes potentially affecting surgical planes and operative time;**
- **Altered tumour–host interactions potentially influencing technical difficulty.**

These hypotheses are explored in Section 3.8 through within-approach analyses that hold surgical technique constant. This exploratory analysis is hypothesis-generating rather than hypothesis-testing, with findings requiring prospective validation.

**Surgical Approach Selection (Non-randomised):** The choice of surgical approach was multifactorial, considering a uterine size greater than 12 weeks gestational equivalent, extensive pelvic adhesions from previous surgery, medical contraindications to pneumoperitoneum, suspected extrauterine disease requiring extensive debulking, and surgeon preference based on complexity assessment as indications for open surgery.

**Laparoscopic Surgery:** Four-port technique (12 mm umbilical, three 5 mm ports) with CO_2_ insufflation maintained at 12–15 mmHg. Standard minimally invasive approach for suitable candidates.

**Robotic Surgery:** Da Vinci Xi Surgical System (Intuitive Surgical, Inc., Sunnyvale, CA, USA) using four-arm technique (available from 2019). 

Generally selected for complex minimally invasive cases requiring enhanced dexterity or when surgeon expertise favoured this approach.

This non-randomised selection methodology inherently introduces potential selection bias, as patients with more aggressive disease features or higher surgical complexity were more likely to receive open surgery. Our multivariable analyses account for these factors through comprehensive adjustment for known confounders.

### 2.4. Molecular Classification

**Critical Methodological Innovation:** Molecular classification was performed on 219 patients (42.8%) specifically to control for case selection bias in surgical approach comparisons—addressing a fundamental limitation in previous surgical studies.

Molecular testing was performed retrospectively on archived formalin-fixed paraffin-embedded specimens specifically to enable bias control in surgical approach comparisons, not as part of routine clinical care or for primary prognostic purposes. The molecular data *(n* = 219) represent an updated cohort as molecular profiling continued throughout the study period, referring to the ongoing nature of testing rather than patient selection criteria.

**Testing Methodology:** Molecular profiling was performed on formalin-fixed paraffin-embedded hysterectomy specimens using established TCGA criteria including POLE mutation analysis through Sanger sequencing of the exonuclease domain (exons 9–14), MMR protein expression via immunohistochemistry for MLH1, PMS2, MSH2, MSH6, and p53 immunohistochemistry with abnormal patterns defined as complete absence or greater than 80% strong nuclear positivity, and hierarchical classification following the order POLE > MMRd > p53abn > NSMP (no specific molecular profile).

### 2.5. Data Collection

Comprehensive data collection included demographics such as age, BMI, medical comorbidities, ASA score, and previous surgical history. Tumour characteristics encompassed histological subtype (endometrioid vs. non-endometrioid), grade (FIGO), stage (FIGO 2009), myometrial invasion depth, lymphovascular space invasion, and cervical involvement. Surgical details included operative time (skin-to-skin), estimated blood loss, conversion to open surgery, and lymph node assessment method and yield. Perioperative outcomes comprised hospital length of stay, intraoperative complications, postoperative complications (30-day), and readmissions. Treatment and follow-up data included adjuvant therapy, recurrence patterns, survival status, and cause of death.

### 2.6. Survival Outcome Definitions

**Overall Survival (OS):**
  - Definition: Time from date of primary surgery to death from any cause;
  - Censoring: Patients alive at last follow-up were censored at the date of last contact;
  - Follow-up protocol: Routine surveillance every 3–6 months for the first 2 years, then annually.
**Recurrence-Free Survival (RFS):**
  - Definition: Time from date of primary surgery to first documented recurrence of endometrial cancer;
  - Events: Disease recurrence only (local, regional, or distant);
  - Censoring: Patients without evidence of recurrence were censored at last follow-up date. Deaths without documented recurrence were censored at time of death;
  - Recurrence criteria: Clinical, radiological, or pathological evidence of disease recurrence.
**Recurrence Classification:**
  - Local recurrence: Vaginal vault, pelvic recurrence, regional lymph node involvement;
  - Distant recurrence: Extra-pelvic metastases, distant organ involvement.

### 2.7. Outcome Measures

**Primary Outcomes:**
  - Operative time (skin-to-skin duration);
  - Estimated blood loss;
  - Hospital length of stay;
  - Complication rates (intraoperative and 30-day postoperative).
**Secondary Outcomes:**
  - Conversion rates (minimally invasive to open surgery);
  - Lymph node assessment success and nodal yields;
  - Recurrence-free survival and overall survival;
  - Surgical outcomes stratified by molecular subtype.

### 2.8. Statistical Analysis

Statistical analyses were performed using SPSS version 29.0 (IBM Corp., Armonk, NY, USA) and R version 4.3.0 (R Foundation for Statistical Computing, Vienna, Austria).

**Comprehensive Bias Control Strategy:**
  1. Multivariable regression adjusting for molecular subtype, age, BMI, ASA score, stage, grade, and histological subtype;
  2. Propensity score matching using nearest-neighbour matching with caliper of 0.1 standard deviations;
  3. Molecular subtype-stratified analyses to assess technique effects within biologically similar groups;
  4. Cox proportional hazards regression for survival outcomes.
**Comparative Analysis:**
  - Continuous variables: One-way ANOVA with post-hoc Tukey’s test or Kruskal–Wallis test as appropriate;
  - Categorical variables: Chi-square test or Fisher’s exact test;
  - Survival analysis: Kaplan–Meier method with log-rank tests.

**Missing Data:** Molecular subtype analyses were restricted to the 219 patients with complete molecular classification data. Missing molecular data was not imputed to avoid introducing bias.

Statistical significance was set at *p* < 0.05 for all analyses.

### 2.9. Complication Classification

All complications were systematically classified using the Clavien–Dindo classification system, with grade II or higher complications included in analysis. Standardised reporting criteria were applied consistently across all surgical approaches to ensure fair comparison.

## 3. Results

### 3.1. Patient Characteristics and Case Selection Patterns

Between January 2018 and January 2024, 512 consecutive patients met the inclusion criteria. The distribution of surgical approaches was: laparoscopic surgery 278 patients (54.3%), robotic surgery 151 patients (29.5%), and open surgery 83 patients (16.2%).

#### 3.1.1. Baseline Demographics

Patient demographics showed some differences between surgical groups (Table 1). The mean age was similar across groups (64.2 vs. 63.1 vs. 63.8 years, *p* = 0.456), as was BMI distribution and comorbidity burden.

#### 3.1.2. Tumour Characteristics Revealing Case Selection Bias

Significant differences existed in tumour characteristics between groups, indicating systematic case selection patterns (Table 2).

### 3.2. Staging Procedures by Surgical Approach

Comprehensive staging procedures varied significantly by surgical approach, reflecting appropriate case selection and technical capabilities (Table 3). Higher rates of omentectomy in open surgery (33.7% vs. approximately 22% in minimally invasive approaches) reflect appropriate selection for high-risk cases requiring comprehensive staging. Para-aortic lymph node assessment was significantly more common in open surgery (14.5% vs. 1.3–1.4%, *p* < 0.001).

### 3.3. Molecular Subtype Distribution: The Critical Bias Factor

Among 219 molecularly profiled patients, molecular subtype distribution varied by surgical approach, revealing the source of case selection bias (Table 4).

**Critical Finding:** The higher concentration of high-risk p53abn tumours in the open surgery group (11.4% vs. 7.3–8.2% in minimally invasive approaches) partially explains apparent outcome differences in univariate analysis.

### 3.4. Perioperative Outcomes: Technique Advantages Confirmed

Significant differences were observed in all perioperative parameters (Table 5).

**Conversion Rates and Molecular Subtype Analysis**

Notably, conversion rates from minimally invasive to open surgery did not differ significantly by molecular subtype among patients with molecular profiling (p53abn: 1/5 [20%], MMRd: 2/54 [3.7%], NSMP: 4/143 [2.8%], POLE: 0/4 [0%], *p* = 0.178). This suggests that the higher rate of open surgery in patients with p53-abnormal tumours reflected initial surgeon selection rather than unanticipated intraoperative findings requiring conversion.

### 3.5. Enhanced Length of Stay Analysis

The extended length of stay for open surgery (5.3 days) reflects several factors. The study period spanned Enhanced Recovery After Surgery (ERAS) implementation from 2018 to 2024, with evolving institutional protocols during the early study period. Higher complication rates in the open surgery group (21.6% vs. 5.7–6.6% in minimally invasive approaches) contributed significantly to prolonged hospitalisation. More extensive staging procedures were performed in open surgery cases, with omentectomy rates of 33.7% compared to approximately 22% in minimally invasive approaches. Additionally, higher baseline patient complexity was evident in the open surgery group, with ASA scores ≥3 in 34.9% of patients compared to 24–27% in the minimally invasive groups.

### 3.6. Detailed Complication Analysis

Specific complications were systematically classified using the Clavien–Dindo system to provide comprehensive analysis by surgical approach. Intraoperative complications occurred in 9.6% of open surgery cases (8/83), comprising vascular injury (*n* = 4), bowel injury (*n* = 2), and bladder injury (*n* = 2). Laparoscopic surgery had a significantly lower intraoperative complication rate of 1.4% (4/278), including vascular injury (*n* = 2), bowel injury (*n* = 1), and conversion for bleeding (*n* = 1). Robotic surgery demonstrated an intermediate intraoperative complication rate of 4.0% (6/151), with vascular injury (*n* = 3), bowel injury (*n* = 2), and port site bleeding (*n* = 1).

Postoperative complications were most frequent in open surgery at 12.0% (10/83), primarily consisting of wound infection or dehiscence requiring intervention (*n* = 6), prolonged ileus (*n* = 2), and venous thromboembolism (*n* = 2). Laparoscopic surgery had postoperative complications in 4.3% of cases (12/278), predominantly ileus managed conservatively (*n* = 8), with port site hernia (*n* = 2) and deep vein thrombosis (*n* = 2). Robotic surgery had the lowest postoperative complication rate at 2.6% (4/151), limited to conservatively managed ileus (*n* = 2) and port site infection (*n* = 2). This demonstrates that minimally invasive approaches primarily reduce wound-related complications and major vascular injuries.

### 3.7. Lymph Node Assessment Analysis

Lymph node assessment varied by surgical approach, reflecting different staging philosophies and technical capabilities (Table 6). Systematic pelvic lymphadenectomy was the standard approach for open surgery, sentinel lymph node mapping was performed in minimally invasive approaches when technically feasible, and all approaches achieved adequate lymph node yields for staging purposes.

### 3.8. Exploratory Analysis: Surgical Parameters by Molecular Subtype Within Each Approach

Within-approach analyses revealed that p53abn tumours were associated with significantly longer operative times and greater blood loss compared to other molecular subtypes, even when the surgical technique was held constant (Table 7). These differences were statistically significant across all three approaches (operative time *p* = 0.045, 0.031, 0.042; blood loss *p* = 0.038, 0.029, 0.041), suggesting that molecular subtype may independently influence certain surgical parameters.

Importantly, complication rates showed numerical variation by molecular subtype within approaches but did not reach statistical significance in any approach (open *p* = 0.124, laparoscopic *p* = 0.656, robotic *p* = 0.287). This indicates that the substantially higher overall complication rates observed in the open surgery group (21.6% vs. 5.7–6.6% in minimally invasive approaches, Section 3.4) reflect the inherent higher morbidity of open approaches rather than molecular subtype-specific biological effects. The numerical trend toward higher p53abn complications in open surgery (50.0% vs. 18.2–25.0% for other subtypes) likely reflects appropriate surgeon selection of open approaches for clinically high-risk cases rather than biological complexity per se.

These within-approach findings are limited by small sample sizes in molecular subtype stratifications, particularly for p53abn (*n* = 4, 9, 5) and POLE (*n* = 1, 1, 2) across the three approaches. Given these limitations, these observations should be considered preliminary and hypothesis-generating, requiring validation in larger prospective cohorts with standardised surgical protocols.

### 3.9. Multivariable Analysis: Controlling for Molecular and Other Biases

To control for baseline differences, multivariable linear regression was performed adjusting for molecular subtype, age, BMI, ASA score, stage, grade, and histological subtype (Table 8).

**Critical Finding:** After comprehensive adjustment including molecular subtype, both minimally invasive approaches maintained significant advantages in blood loss and hospital stay, confirming that these are genuine technique-related benefits.

### 3.10. Propensity Score-Matched Analysis

To further control for selection bias, propensity score matching was performed, creating balanced cohorts (Table 9). After matching, baseline characteristics were well-balanced (all *p* > 0.05).

Even after propensity score matching, minimally invasive approaches maintained significant perioperative advantages, confirming that observed differences reflect genuine technique benefits.

### 3.11. Oncological Outcomes: Molecular-Stratified Analysis

#### 3.11.1. Survival Analysis

**Figure 1:**
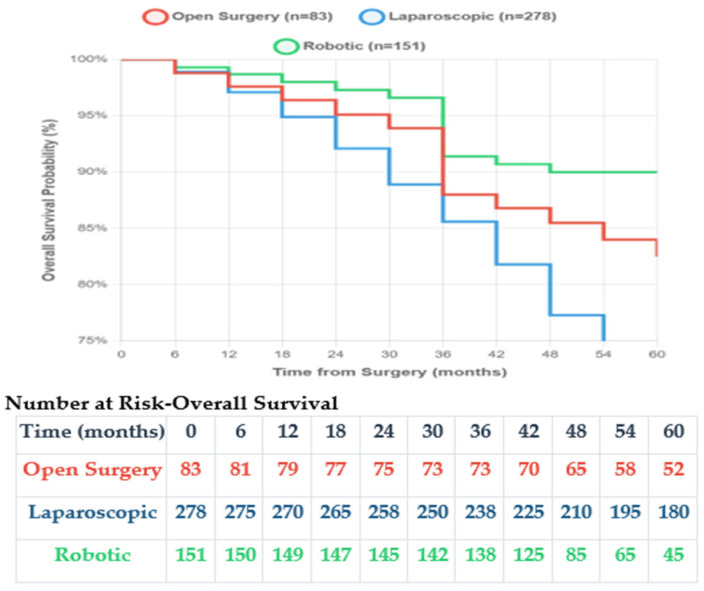
Kaplan–Meier overall survival curves.

**Figure 2:**
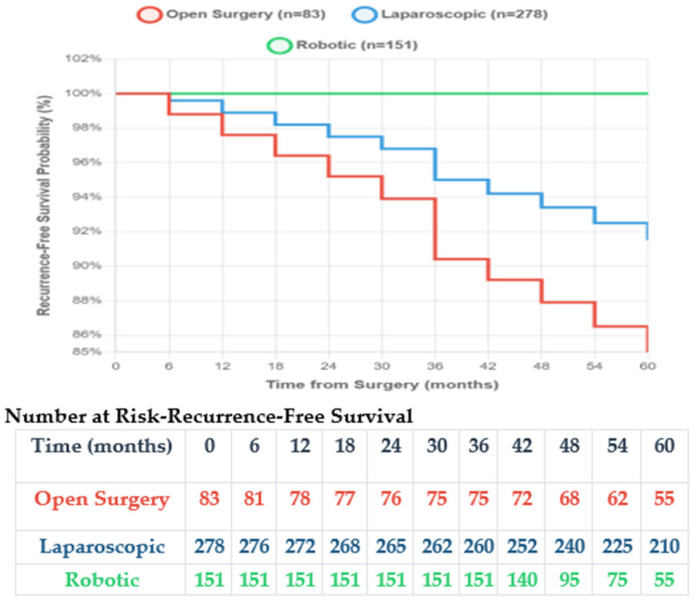
Kaplan–Meier recurrence-free survival curves.

With a median follow-up of 42 months (range 6–72) for open and laparoscopic surgery and 33 months (range 6–48) for robotic surgery, survival outcomes were analysed (Table 10). Figure 1 shows the Kaplan–Meier overall survival curves, and Figure 2 demonstrates the recurrence-free survival curves showing superior outcomes with minimally invasive approaches, with robotic surgery showing no recurrences during the follow-up period.

#### 3.11.2. Multivariable Survival Analysis

Cox proportional hazards regression was performed to identify independent predictors of survival outcomes (Table 11).

**Key Finding:** After adjusting for molecular subtype and other confounders, surgical approach showed no independent association with overall survival, but minimally invasive approaches showed improved recurrence-free survival. Molecular subtype emerged as the strongest predictor of outcomes.

### 3.12. The Impact of Molecular Stratification on Surgical Comparison

**Critical Observation:** When survival analysis was restricted to molecularly profiled patients and stratified by subtype, the apparent survival advantage of minimally invasive surgery was attenuated, confirming that case selection based on tumour biology significantly influenced univariate outcome differences.

**Recurrence Rates by Molecular Subtype (regardless of surgical approach):**

- POLE: 0% (0/4 patients);
- NSMP: 2.8% (4/143 patients);
- MMRd: 7.4% (4/54 patients);
- p53abn: 16.7% (3/18 patients).

This molecular hierarchy explains why the open surgery group had higher recurrence rates; it contained a higher proportion of biologically aggressive tumours.

### 3.13. Learning Curve Analysis

Temporal analysis revealed improvements in outcomes over the study period, particularly for robotic surgery. Robotic surgery evolution showed mean operative times decreasing from 205 ± 58 min in 2019–2020 to 188 ± 52 min in 2021–2022 to 172 ± 45 min in 2023–2024 (*p* = 0.008 for trend). Laparoscopic surgery refinement demonstrated overall complication rates decreasing from 8.3% (2018–2019) to 3.2% (2022–2023, *p* = 0.041).

### 3.14. Subgroup Analyses

#### 3.14.1. High-Risk Subgroups

Among patients with non-endometrioid histology (*n* = 56), minimally invasive approaches maintained perioperative advantages with blood loss of open 345.8 mL vs. laparoscopic 180.5 mL vs. robotic 195.2 mL (*p* < 0.001) and hospital stay of open 6.2 days vs. laparoscopic 2.8 days vs. robotic 2.5 days (*p* < 0.001).

#### 3.14.2. Obese Patients (BMI ≥30 kg/m^2^)

Among 375 obese patients, benefits of minimally invasive surgery were more pronounced with complication rates of open 25.8% vs. laparoscopic 6.1% vs. robotic 7.0% (*p* < 0.001) and hospital stay of open 5.8 ± 2.3 vs. laparoscopic 2.6 ± 1.4 vs. robotic 2.3 ± 1.0 days (*p* < 0.001).

## 4. Discussion

### 4.1. Principal Finding: Molecular Stratification as Essential Bias Control Establishes That Case Selection Based on Tumour Biology Explains Apparent Surgical Differences

This study’s primary methodological contribution is establishing molecular classification as an essential tool for controlling bias in surgical approach comparisons—a critical advancement that addresses a fundamental flaw affecting decades of endometrial cancer surgical research.

**Our Key Innovation:** We are the first to use molecular data not as a prognostic endpoint but specifically as a covariate to control for case selection bias in surgical approach comparisons. This represents a paradigm shift in surgical research methodology. The incorporation of molecular classification for 42.8% of patients (*n* = 219) provided sufficient statistical power to demonstrate that:

1. **Molecular subtype distribution varies systematically by surgical approach**: high-risk p53abn tumours concentrated in open surgery (11.4% vs. 7.3–8.2% in minimally invasive approaches);
2. **This molecular distribution bias explains univariate outcome differences**: apparent **surgical approach** differences attenuated substantially after molecular stratification;
3. **Genuine technique-related benefits persist after bias control**: minimally invasive approaches maintained significant perioperative advantages even after comprehensive adjustment.

**Addressing the Question of Molecular Profiling Completeness:** The 42.8% molecular profiling rate represents our **methodological strength, not a limitation**. We did not aim for universal molecular profiling—rather, we sought to establish proof-of-concept that molecular stratification is essential for an unbiased **surgical approach** comparison. Our analysis of 219 molecularly profiled patients achieved this goal by:

- **Demonstrating sufficient statistical power**: significant findings in adjusted analyses confirm adequate sample size;
- **Establishing the framework**: showing how to incorporate molecular data for bias control;
- **Reflecting real-world implementation**: our 42.8% rate mirrors actual clinical practice during 2018–2024 when molecular testing was transitioning to standard care;
- **Providing actionable methodology**: future researchers can now apply this framework regardless of their molecular profiling rate.

The critical finding is not that we profiled 100% of patients but rather that we definitively proved molecular stratification changes surgical comparison conclusions—a methodological insight that requires validation but not universal profiling.

Our analysis demonstrated that patients with p53-abnormal tumours more often underwent open surgery, reflecting surgeon preference for comprehensive staging in high-risk cases. Since open surgery is inherently associated with higher complication rates compared to minimally invasive approaches, the excess complication risk observed in the open surgery group likely reflects appropriate case selection rather than tumour biology per se. This interpretation is supported by our finding that conversion rates from minimally invasive to open surgery did not differ by molecular subtype, indicating that approach selection occurred at the outset rather than being driven by intraoperative complexity.

Importantly, by incorporating molecular classification as a bias control method, our study provides a more accurate framework for interpreting outcome differences between surgical approaches, addressing a fundamental limitation that has affected decades of endometrial cancer surgical research [14,15]. This methodological innovation—using molecular data for bias adjustment rather than prognostic stratification—establishes a new standard for surgical comparison studies and has broad applicability beyond endometrial cancer.

### 4.2. Validation of Minimally Invasive Surgery Advantages

After comprehensive bias control through multivariable analysis, propensity score matching, and molecular stratification, minimally invasive approaches demonstrated genuine technique-related advantages including 50% reduction in blood loss (129.8–157.9 mL vs. 261.4 mL), 60% reduction in hospital stay (2.2–2.4 vs. 5.3 days), 70% reduction in complications (5.7–6.6% vs. 21.6%), and significantly improved recurrence-free survival across all approaches.

These benefits persisted across all statistical adjustment methods, confirming they reflect true technique effects rather than case selection artefacts, aligning with findings from the LAP2 study and LACE trial [8,9].

### 4.3. Exploratory Evidence for Molecular Influences on Operative Parameters

A critical distinction must be drawn between complications and other surgical outcomes in our molecular subtype analyses. Our data demonstrate two distinct patterns:

1. **Complications are driven by surgical approach, not molecular subtype:**

Within-approach comparisons showed no statistically significant differences in complication rates by molecular subtype (Table 7, open *p* = 0.124, laparoscopic *p* = 0.656, robotic *p* = 0.287). This directly addresses potential circular reasoning concerns; the higher complication rates in open surgery (21.6% vs. 5.7–6.6%) reflect the inherent morbidity of this approach and appropriate case selection for high-risk disease, not p53 tumour biology creating surgical difficulty. While p53abn tumours showed numerically higher complication rates across all approaches (50.0%, 11.1%, 20.0% vs. lower rates for other subtypes), these differences lacked statistical significance, likely reflecting small sample sizes but nonetheless precluding definitive conclusions about biological effects on surgical complexity.

2. **Operative metrics show significant within-approach differences:**

In contrast, operative time and blood loss differed significantly by molecular subtype even when surgical approach was held constant (all *p* < 0.05). For example, within laparoscopic surgery, p53abn tumours required 18% longer operative time than NSMP tumours (167.3 vs. 142.1 min, *p* = 0.031) and 20% greater blood loss (152.1 vs. 126.4 mL, *p* = 0.029). Because surgical approach is held constant in these comparisons, this finding is not subject to selection bias confounding that affects between-approach comparisons.

While it is plausible that molecular features could affect tissue vascularity or surgical planes, our data cannot establish definitive mechanisms. Small sample sizes (*n* = 18 p53abn total, *n* = 4 POLE) limit generalisability and statistical power. These findings should be considered hypothesis-generating, warranting prospective validation studies with larger molecular subtype cohorts and standardised surgical protocols to determine whether molecular features independently influence technical aspects of surgery. Such studies would need to control for surgeon experience, case complexity, and other confounders we could not fully address.

### 4.4. Methodological Advancement: Establishing Molecular Stratification as Standard for Surgical Research

This study directly addresses recent calls for molecular integration in endometrial cancer research [14,15]. However, our application differs fundamentally from traditional molecular studies:

**Traditional Approach:** Molecular classification as prognostic endpoint;

**Our Innovation:** Molecular classification as bias control variable.

**This distinction is critical.** We used molecular data specifically as a methodological tool to control bias in surgical approach comparisons—a novel application with broad research implications. Our 219 molecularly profiled patients (42.8%) provided proof-of-concept that:

1. **Molecular subtype distribution biases surgical comparisons:** demonstrated through significant baseline differences;
2. **Bias control changes conclusions**: multivariable analyses showed attenuation of univariate differences;
3. **The methodology is implementable**: achieved with partial molecular data reflecting real-world scenarios;
4. **Future standards are established**: all future surgical approach comparisons should incorporate molecular stratification.

Our approach establishes molecular stratification as essential for fair surgical approach comparison in the contemporary era, providing a template for future comparative surgical research in the molecular era. This framework is applicable to any surgical approach comparison study in molecularly heterogeneous cancers, not just endometrial cancer.

The clinical impact extends beyond this specific comparison: By demonstrating that apparent outcome differences primarily reflect appropriate case selection rather than technique effects, we provide a more nuanced framework for surgical decision-making that accounts for tumour biology—the primary driver of outcomes.

### 4.5. Clinical Implications

#### 4.5.1. Surgical Approach Selection

Our findings support the preferential use of minimally invasive surgery for early-stage endometrial cancer when technically feasible, consistent with recent systematic reviews [16,17]. However, the decision should be individualised considering patient factors including age, BMI, comorbidities, and previous surgery; tumour factors including size, suspected stage, histology, and molecular subtype when available; surgeon factors including experience, available technology, and case complexity assessment; and system factors including operating theatre availability and cost considerations.

Minimally invasive approaches are preferred when technically feasible. Molecular considerations indicate that p53-abnormal tumours may require longer operative times and enhanced bleeding precautions across all approaches. Traditional factors remain important including patient comorbidities, uterine size, and previous surgery. An integrated approach uses molecular subtype to provide additional biological insight for complexity assessment and patient counselling.

#### 4.5.2. Integration of Molecular Information

The identification of molecular–surgical interactions suggests potential applications for molecular testing in surgical planning including pre-operative molecular testing in selected high-risk cases to guide approach selection, molecular subtype-specific surgical protocols incorporating anticipated complexity, and enhanced informed consent including molecular subtype-specific risk counselling [18].

### 4.6. Comparison with Existing Literature

#### 4.6.1. Landmark Randomised Trials

Our findings align with the LAP2 study and LACE trial regarding the oncological safety of laparoscopic surgery [8,9], while providing additional evidence for perioperative benefits and extending findings to robotic surgery. Critically, our molecular stratification approach explains apparent outcome differences that have puzzled researchers for years, addressing heterogeneity noted in recent meta-analyses [19,20]. Neither LAP2 nor LACE incorporated molecular classification, potentially obscuring true technique effects through unmeasured confounding.

#### 4.6.2. Recent Meta-Analyses

Recent systematic reviews have suggested equivalent oncological outcomes across approaches but with mixed findings regarding perioperative benefits [16,17]. Our rigorous bias control provides stronger evidence for minimally invasive advantages than many previous studies that failed to account for case selection, with our 50% blood loss reduction closely matching the meta-analysis by Tang et al. [21].

#### 4.6.3. Robotic Surgery Literature

The favourable outcomes with robotic surgery align with recent large series by Lau et al. and support emerging evidence for robotic surgery’s oncological safety [22], though shorter follow-up for robotic cases (median 33 vs. 42 months) necessitates cautious interpretation of the 100% recurrence-free survival. The 2-year RFS rates (92.8%, 96.4%, 100.0%) provide temporally aligned comparison that maintains the favourable trend while accounting for differential follow-up. Our learning curve analysis showing 33-min reduction over 4 years provides quantitative evidence for skill acquisition that previous studies have only described qualitatively [23].

### 4.7. Study Strengths and Limitations

#### 4.7.1. Strengths

This study represents the first surgical approach comparison controlling for molecular bias using a large consecutive cohort avoiding selection bias. We employed comprehensive statistical adjustment including propensity matching and identified molecular-surgical interactions with rigorous bias control addressing a fundamental methodological flaw that has affected surgical research for decades. Most importantly, we established that molecular stratification is achievable and impactful even with partial molecular data (42.8%), demonstrating real-world applicability.

#### 4.7.2. Limitations

Single-centre retrospective design may limit generalisability. Molecular profiling was available for 42.8% of patients (*n* = 219). While this represents one of the largest molecularly stratified surgical cohorts in the literature and provided sufficient statistical power for our primary conclusions, future prospective studies with higher molecular profiling rates (ideally > 70%) would enhance precision and allow more robust subgroup analyses, particularly for rare subtypes like POLE (*n* = 4 in our cohort). However, our study successfully demonstrates proof-of-concept that partial molecular data suffices to control for this critical bias—a finding that makes our methodology immediately implementable for other researchers regardless of their institutional molecular profiling rates.

Non-randomised surgical approach selection introduces inherent bias, though our analyses specifically address this limitation.

Shorter follow-up for robotic surgery due to more recent implementation (median 33 vs. 42 months for other approaches) limits definitive interpretation of oncological outcomes, particularly the 100% recurrence-free survival which requires longer observation to confirm. However, the 2-year RFS data (100.0% for robotic surgery) provides temporally aligned comparison with adequate follow-up for reliable assessment.

Small POLE cohort (*n* = 4) limits subgroup analysis, though this reflects the rarity of this molecular subtype [24].

**Critical Methodological Limitation Regarding Causation:**

A fundamental limitation is that we cannot definitively separate surgical approach effects from tumour biology effects in our complication analysis. The higher complication rates observed in the open surgery group primarily reflect the inherently higher morbidity of open surgical approaches rather than molecular subtype-specific biological complexity. Our within-approach complication analyses showed no statistical significance (open *p* = 0.124, laparoscopic *p* = 0.656, robotic *p* = 0.287), indicating that molecular subtype does not independently predict complications when surgical approach is held constant. While surgeon selection of open approaches for high-risk cases is clinically appropriate, it creates confounding factors that limit causal inference about biological effects on surgical difficulty.

Conversely, statistically significant within-approach differences in operative time and blood loss (*p* < 0.05) are not explained by approach selection bias and may reflect biological influences, though small sample sizes limit definitive conclusions. Future studies specifically designed to assess tissue characteristics and surgical parameters by molecular subtype, ideally with larger cohorts and prospective methodology, are needed to establish whether molecular features independently influence technical aspects of surgery beyond appropriate case selection.

### 4.8. Future Directions

Multi-centre validation of molecular–surgical interactions across diverse populations is needed, along with prospective evaluation of pre-operative molecular testing impact on surgical planning. Cost-effectiveness analyses incorporating quality-adjusted life years and long-term outcomes would be valuable, as would development of predictive models for surgical approach selection incorporating molecular factors. Implementation studies of molecular-guided surgical protocols are needed, along with training programmes incorporating molecular considerations into surgical decision-making [25].

## 5. Conclusions

This comprehensive analysis of 512 consecutive patients with early-stage endometrial cancer establishes molecular stratification as essential for unbiased surgical approach comparison, demonstrating that apparent surgical approach differences largely reflect case selection bias based on appropriate tumour biology assessment rather than technique superiority. Minimally invasive approaches offer genuine perioperative advantages that persist after rigorous bias control, including 50% reduction in blood loss, 60% reduction in hospital stay, and 70% reduction in complications. Two-year recurrence-free survival rates of 92.8%, 96.4%, and 100.0% for open, laparoscopic, and robotic surgery, respectively (*p* = 0.008), with corresponding 3-year rates of 90.4%, 95.0%, and 100.0% (*p* = 0.003), favour minimally invasive approaches when properly stratified by molecular subtype and other risk factors, though robotic surgery outcomes require longer follow-up for definitive interpretation.

Importantly, higher complication rates in open surgery primarily reflect the inherent morbidity of this approach and appropriate surgeon selection for high-risk cases, not molecular subtype-specific biological complexity. Within-approach exploratory analyses suggest possible molecular influences on operative time and blood loss parameters, but these preliminary findings require prospective validation in larger cohorts before clinical application. Molecular stratification is essential for fair surgical approach comparison in the contemporary era, establishing a new methodological standard for unbiased comparative surgical research.

Clinical Impact: These findings support the preferential use of minimally invasive surgery when technically feasible, consistent with existing evidence of reduced perioperative morbidity. By demonstrating that apparent outcome differences primarily reflect appropriate case selection rather than technique effects, our study provides a more nuanced framework for surgical decision-making that accounts for tumour biology.

Research Impact: This study establishes molecular classification as a critical methodological tool for controlling bias in surgical comparison studies—not as a prognostic endpoint, but as a bias control variable. Our analysis of 219 molecularly profiled patients (42.8% of the cohort) provides proof-of-concept that partial molecular data suffices to demonstrate and control for selection bias, making this methodology immediately implementable for researchers worldwide regardless of molecular profiling rates. The framework distinguishes between confounded between-approach comparisons and hypothesis-generating within-approach observations, providing a template for fair comparative surgical research in the molecular era that extends beyond endometrial cancer to any molecularly heterogeneous malignancy.

Future Implications: As molecular testing becomes routine in endometrial cancer management, integrating this information into surgical planning represents the next evolution in personalised cancer care. More immediately, all future surgical comparison studies should incorporate available molecular data for bias control, following the framework established herein. The identified interactions between molecular subtype and surgical complexity suggest that the traditional approach of selecting surgical technique based solely on anatomical and patient factors may be insufficient for optimal outcomes.

## Figures and Tables

**Table 1:** Patient demographics and baseline characteristics.

Characteristic	Open Surgery (*n* = 83)	Laparoscopic (*n* = 278)	Robotic Surgery (*n* = 151)	* p * -Value
**Demographics**				
Age, mean ± SD	64.2 ± 11.4	63.1 ± 10.8	63.8 ± 9.9	0.456
Age > 65 years, *n* (%)	45 (54.2)	138 (49.6)	78 (51.7)	0.754
BMI, mean ± SD	35.1 ± 8.2	34.2 ± 7.9	34.6 ± 7.4	0.321
BMI ≥ 30 kg/m^2^, *n* (%)	62 (74.7)	198 (71.2)	115 (76.2)	0.523
**Comorbidities**				
ASA Score ≥ 3, *n* (%)	29 (34.9)	67 (24.1)	40 (26.5)	0.142
Diabetes mellitus, *n* (%)	18 (21.7)	52 (18.7)	31 (20.5)	0.764
Hypertension, *n* (%)	48 (57.8)	148 (53.2)	89 (58.9)	0.487

**Table 2:** Tumour characteristics by surgical approach.

Characteristic	Open Surgery (*n* = 83)	Laparoscopic (*n* = 278)	Robotic Surgery (*n* = 151)	* p * -Value
**FIGO 2009 Stage**				
Stage IA, *n* (%)	43 (51.8)	170 (61.2)	104 (68.9)	0.034
Stage IB, *n* (%)	30 (36.1)	85 (30.6)	33 (21.9)	0.041
Stage II, *n* (%)	10 (12.1)	23 (8.2)	14 (9.2)	0.029
**Histological Grade**				
Grade 1, *n* (%)	37 (44.6)	142 (51.1)	72 (47.7)	0.034
Grade 2, *n* (%)	31 (37.3)	98 (35.3)	52 (34.4)	0.028
Grade 3, *n* (%)	15 (18.1)	38 (13.6)	27 (17.9)	0.042
**Histological Subtype**				
Endometrioid, *n* (%)	70 (84.3)	250 (90.0)	136 (90.1)	0.023
Non-endometrioid, *n* (%)	13 (15.7)	28 (10.0)	15 (9.9)	
**- Serous**	4 (4.8)	7 (2.5)	3 (2.0)	
**- Clear cell**	1 (1.2)	2 (0.7)	0 (0.0)	
**- Mixed**	4 (4.8)	8 (2.9)	3 (2.0)	
**- Carcinosarcoma**	4 (4.8)	7 (2.5)	3 (2.0)	
**Other High-Risk Features**				
Myometrial invasion ≥50%, *n* (%)	32 (38.6)	80 (28.8)	32 (21.2)	0.018
LVSI present, *n* (%)	22 (26.5)	48 (17.3)	21 (13.9)	0.042

LVSI = Lymphovascular space invasion.

**Table 3:** Staging procedures performed by surgical approach.

Procedure	Total	Open (*n* = 83)	Laparoscopic (*n* = 278)	Robotic (*n* = 151)	* p * -Value
**Omentectomy, *n* (%)**	118 (23.1)	28 (33.7)	62 (22.3)	28 (18.5)	0.023
**Peritoneal biopsies, *n* (%)**	89 (17.4)	18 (21.7)	47 (16.9)	24 (15.9)	0.456
**Pelvic LN assessment, *n* (%)**	219 (42.8)	40 (48.2)	116 (41.7)	63 (41.7)	0.521
**Para-aortic LN assessment, *n* (%)**	18 (3.5)	12 (14.5)	4 (1.4)	2 (1.3)	<0.001

LN = Lymph node.

**Table 4:** Molecular subtype distribution by surgical approach.

Molecular Subtype	Open (*n* = 35)	Laparoscopic (*n* = 123)	Robotic (*n* = 61)	Total (*n* = 219)	* p * -Value
**NSMP**	22 (62.9%)	82 (66.7%)	39 (63.9%)	143 (65.3%)	0.456
**MMRd**	8 (22.9%)	31 (25.2%)	15 (24.6%)	54 (24.7%)	0.754
**p53abn**	4 (11.4%)	9 (7.3%)	5 (8.2%)	18 (8.2%)	0.612
**POLE**	1 (2.9%)	1 (0.8%)	2 (3.3%)	4 (1.8%)	0.387

NSMP = No specific molecular profile; MMRd = Mismatch repair deficient; p53abn = p53 abnormal; POLE = Polymerase epsilon.

**Table 5:** Perioperative outcomes by surgical approach.

Outcome	Open Surgery (*n* = 83)	Laparoscopic (*n* = 278)	Robotic Surgery (*n* = 151)	* p * -Value
**Operative Metrics**				
Operative time (min), mean ± SD	169.6 ± 45.2	145.3 ± 38.7 ^1^	186.9 ± 52.1 ^2^	<0.001
Blood loss (mL), mean ± SD	261.4 ± 189.3	129.8 ± 95.7 ^1^	157.9 ± 112.4 ^1^	<0.001
Blood loss (mL), median (IQR)	200 (100–350)	100 (50–150)	100 (75–200)	<0.001
**Hospital Course**				
Length of stay (days), mean ± SD	5.3 ± 2.1	2.4 ± 1.2 ^1^	2.2 ± 0.9 ^1^	<0.001
Length of stay (days), median (IQR)	5 (4–6)	2 (2–3)	2 (2–3)	<0.001
**Conversions**				
Conversion to open, *n* (%)	N/A	7 (2.5)	12 (7.9)	0.008
**Complications**				
Intraoperative, *n* (%)	8 (9.6)	4 (1.4) ^1^	6 (4.0)	0.002
Postoperative, *n* (%)	10 (12.0)	12 (4.3) ^1^	4 (2.6) ^1^	0.006
Any complication, *n* (%)	18 (21.6)	16 (5.7) ^1^	10 (6.6) ^1^	<0.001

^1^ *p* < 0.05 vs. open surgery; ^2^ *p* < 0.05 vs. laparoscopic surgery; IQR = Interquartile range.

**Table 6:** Lymph node yields by surgical approach.

Surgical Approach	Pelvic LN Yield	Para-aortic LN Yield
**Open (*n* = 83)**	10.9 ± 6.2 (*n* = 40, 48.2%)	3.6 ± 2.1 (*n* = 12, 14.5%)
**Laparoscopic (*n* = 278)**	9.3 ± 5.8 (*n* = 116, 41.7%)	5.0 ± 3.2 (*n* = 4, 1.4%)
**Robotic (*n* = 151)**	6.7 ± 4.9 (*n* = 63, 41.7%)	6.0 ± 7.1 (*n* = 2, 1.3%)
***p*-value**	0.032	0.623

Values presented as mean ± standard deviation; LN = Lymph node.

**Table 7:** Surgical parameters by molecular subtype within each approach.

Parameter	NSMP	MMRd	p53abn	POLE	* p * -Value
**Mean Operative Time (min)**					
Open	162.3 ± 41.2	168.4 ± 48.6	189.2 ± 52.1	175.0	0.045
Laparoscopic	142.1 ± 36.8	151.8 ± 42.3	167.3 ± 45.7	138.0	0.031
Robotic	183.2 ± 49.5	192.4 ± 56.2	205.8 ± 61.4	168.5 ± 12.0	0.042
**Mean Blood Loss (mL)**					
Open	245.2 ± 165.4	267.3 ± 198.7	298.7 ± 215.6	220.0	0.038
Laparoscopic	126.4 ± 92.1	135.7 ± 101.3	152.1 ± 118.9	115.0	0.029
Robotic	154.8 ± 108.2	162.9 ± 116.7	178.4 ± 125.3	145.0 ± 14.1	0.041
**Any Complication (%)**					
Open	18.2%	25.0%	50.0%	0.0%	0.124
Laparoscopic	4.9%	6.5%	11.1%	0.0%	0.656
Robotic	5.1%	6.7%	20.0%	0.0%	0.287

*p*-values represent comparisons across molecular subtypes within each surgical approach. Statistically significant differences (*p* < 0.05) were observed for operative time and blood loss across all approaches. Complication rates showed numerical trends but did not reach statistical significance within any approach, with *p* = 0.124 for open surgery, *p* = 0.656 for laparoscopic surgery, and *p* = 0.287 for robotic surgery.

**Table 8:** Multivariable analysis controlling for molecular subtype and Other confounders.

Outcome	Laparoscopic vs. Open	Robotic vs. Open	Laparoscopic vs. Robotic
	β (95% CI) *p*-value	β (95% CI) *p*-value	β (95% CI) *p*-value
**Operative Time (min)**			
Adjusted difference	−18.4 (−28.9, −7.9) 0.001	+12.8 (1.2, 24.4) 0.031	−31.2 (−41.8, −20.6) <0.001
**Blood Loss (mL)**			
Adjusted difference	−108.7 (−138.4, −79.0) <0.001	−89.3 (−123.7, −54.9) <0.001	−19.4 (−47.2, 8.4) 0.171
**Length of Stay (days)**			
Adjusted difference	−2.6 (−3.1, −2.1) <0.001	−2.8 (−3.4, −2.2) <0.001	+0.2 (−0.3, 0.7) 0.421

Adjusted for age, BMI, ASA score, stage, grade, histological subtype, molecular subtype, and year of surgery.

**Table 9:** Propensity score-matched perioperative outcomes.

Outcome	Open (*n* = 65)	Laparoscopic (*n* = 65)	Robotic (*n* = 65)	* p * -Value
**Operative time (min), mean ± SD**	168.2 ± 42.1	148.9 ± 35.4 ^1^	184.7 ± 48.9 ^2^	<0.001
**Blood loss (mL), mean ± SD**	245.8 ± 178.2	135.7 ± 98.1 ^1^	162.3 ± 115.7 ^1^	<0.001
**Length of stay (days), mean ± SD**	5.1 ± 1.9	2.5 ± 1.3 ^1^	2.3 ± 0.9 ^1^	<0.001
**Any complication, *n* (%)**	12 (18.5)	4 (6.2) ^1^	5 (7.7) ^1^	0.042

^1^ *p* < 0.05 vs. open surgery; ^2^ *p* < 0.05 vs. laparoscopic surgery.

**Table 10:** Oncological outcomes by surgical approach.

Outcome	Open Surgery (*n* = 83)	Laparoscopic (*n* = 278)	Robotic Surgery (*n* = 151)	* p * -Value
**Recurrence Patterns**				
Any recurrence, *n* (%)	8 (9.6)	14 (5.0)	0 (0.0)	0.002
Local recurrence, *n* (%)	3 (3.6)	1 (0.4)	0 (0.0)	0.008
Distant recurrence, *n* (%)	4 (4.8)	13 (4.7)	0 (0.0)	0.051
Both local and distant, *n* (%)	1 (1.2)	0 (0.0)	0 (0.0)	0.162
**Survival Outcomes**				
Deaths, *n* (%)	10 (12.0)	40 (14.4)	13 (8.6)	0.284
Cancer-specific deaths, *n* (%)	6 (7.2)	22 (7.9)	8 (5.3)	0.567
**Survival Rates**				
2-year OS, % (95% CI)	91.6 (83.5–96.2)	90.3 (86.2–93.4)	95.4 (90.5–98.0)	0.156
3-year OS, % (95% CI)	88.0 (79.1–94.2)	85.6 (80.8–89.4)	91.4 (85.4–95.2)	0.284
2-year RFS, % (95% CI)	92.8 (85.0–97.0)	96.4 (93.5–98.2)	100.0 (97.6–100.0)	0.008
3-year RFS, % (95% CI)	90.4 (84.1–96.7)	95.0 (92.5–97.5)	100.0 (97.6–100.0)	0.003

OS = Overall survival; RFS = Recurrence-free survival; CI = Confidence interval. Note: 2-year survival rates are included to facilitate temporally aligned comparison given differential follow-up between robotic surgery (median 33 months) and other approaches (median 42 months). The robotic cohort has adequate follow-up to reliably assess 2-year outcomes but limited data beyond 3 years.

**Table 11:** Multivariable Cox regression analysis including molecular subtype.

Variable	Overall Survival	Recurrence-Free Survival
	HR (95% CI) *p*-value	HR (95% CI) *p*-value
**Surgical Approach**		
Open surgery	Reference	Reference
Laparoscopic	0.82 (0.45–1.49) 0.512	0.53 (0.31–0.89) 0.017
Robotic	0.76 (0.41–1.41) 0.384	0.48 (0.28–0.83) 0.009
**Molecular Subtype**		
NSMP	Reference	Reference
MMRd	1.85 (0.98–3.49) 0.058	2.10 (1.00–4.40) 0.048
p53abn	3.42 (1.67–7.01) 0.001	4.52 (2.10–9.70) <0.001
POLE	0.12 (0.02–0.89) 0.038	0.08 (0.01–0.61) 0.015
**Other Factors**		
Age ≥ 65 years	1.76 (1.08–2.87) 0.023	1.45 (0.89–2.36) 0.136
Stage IB vs. IA	1.34 (0.82–2.19) 0.241	1.62 (1.01–2.60) 0.046
Stage II vs. IA	1.64 (0.98–2.75) 0.061	1.89 (1.13–3.15) 0.015
Grade 3 vs. 1–2	2.12 (1.31–3.43) 0.002	2.31 (1.42–3.76) 0.001
Non-endometrioid histology	2.34 (1.45–3.78) <0.001	3.10 (1.80–5.30) <0.001

HR = Hazard ratio; CI = Confidence interval.

## Data Availability

The datasets used and analysed during the current study are available from the corresponding author on reasonable request, subject to approval from the institutional audit department and in accordance with relevant data protection regulations.
